# Giant True Lipoma Resection in the Inguinal Canal

**DOI:** 10.7759/cureus.48829

**Published:** 2023-11-15

**Authors:** Edilson Araujo Sabino, Vítor Mamoru Haida, Mateus Colhado Ferreira, Elizabeth Amann Simões, João Marcus Kalil Theezolin

**Affiliations:** 1 Surgery, Complexo Hospitalar do Trabalhador, Curitiba, BRA; 2 General Surgery, Complexo Hospitalar do Trabalhador, Curitiba, BRA; 3 Medicine, Universidade Positivo, Curitiba, BRA

**Keywords:** giant lipoma resection, inguinal hernioplasty, combined surgical technique, inguinal canal, true lipoma

## Abstract

The inguinal canal lipoma, known as spermatic cord lipoma in men or round ligament lipoma in women, has a variable incidence (22.5% to 75%) during inguinal hernioplasty procedures. The presence of a true lipoma in this region is considered rare and often underestimated by surgeons.

A young female patient was diagnosed with a large true inguinal canal lipoma. Resection was performed using both videolaparoscopic and conventional techniques, based on a careful preoperative evaluation of anatomical parameters.

The high incidence of lipomas in the inguinal canal contributes, in part, to the interpretation of fatty masses as "lipomas" during herniorrhaphy procedures. However, many of these are actually extrusions of extraperitoneal adipose tissue, maintaining dimensions within the physiological limits of the region. This confusion in classification highlights the complexity of differentiating between true lipomas and adipose protrusions.

Based on a case report enriched with distinct clinical features and images, we sought to exemplify a surgical approach to a large true inguinal canal lipoma. This report not only emphasizes the rarity of the pathology but also underscores the importance of an effective and differentiated surgical approach for true lipomas in this location.

## Introduction

The incidence of lipomas in the inguinal canal, identified during inguinal hernioplasty procedures, reveals notable variability in studies, spanning a wide range from 22.5% to 75%. This disparity is attributed to the interpretation of fatty masses in the inguinal canal region as "lipomas" in surgical literature [1,2].

However, it is crucial to emphasize that the majority of these occurrences are, in reality, protrusions of adipose tissue in the groin region. This underscores the critical importance of distinguishing these cases from true lipomas in the inguinal canal. Identifying a genuine lipoma in this location is considered uncommon, presenting a significant diagnostic challenge, especially when considering the potential coexistence of an inguinal hernia. The complexity of this differentiation is accentuated by the fact that both conditions share similar clinical symptoms and present similar physical findings.

This similarity can lead to misguided diagnoses, resulting in inappropriate therapeutic approaches [3,4]. In this context, the present report highlights the critical relevance of accurate differentiation between lipomas and non-lipomatous adipose protrusions in the inguinal canal. The confusion between these conditions not only compromises diagnostic accuracy but also leads to inadequate therapeutic approaches.

## Case presentation

A 30-year-old female patient, diagnosed with Grade I obesity, initiated her clinical presentation approximately three years ago, manifesting progressive inguinal discomfort and the presence of a palpable mass measuring approximately 5 centimeters. Initial investigation through ultrasound (US) revealed a left indirect inguinal hernia, characterized as irreducible and containing adipose components.

Throughout the third year of symptom progression, the patient described a significant clinical deterioration with an exponential increase in local volume, independent of Valsalva maneuvers. She reported intense pain and a gradual inability to maintain an orthostatic position. The patient had not sought care, and when she did, she was not operated on because the team that had treated her initially preferred to refer her to a specialized service. On physical examination, there was the presence of a voluminous left inguinal hernia with a lipomatous consistency, extending notably to the left labia majora.

**Figure 1:**
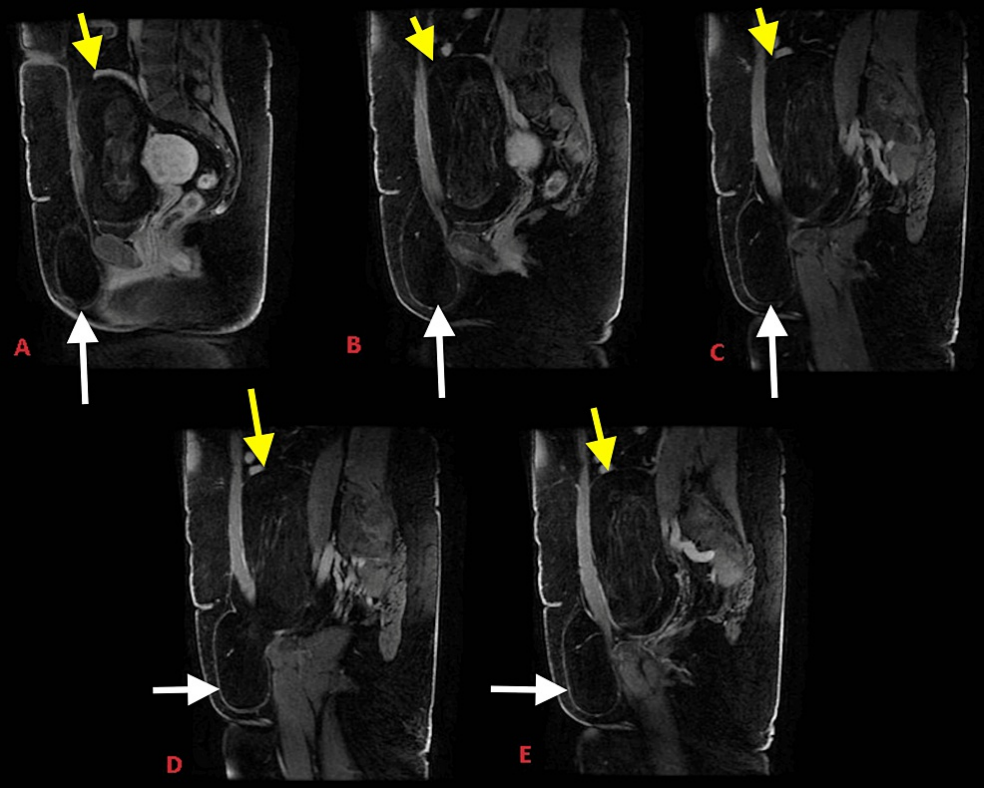
Abdomen and pelvis MRI. Sequential sagittal sections in the contrast-enhanced phase. White arrows point to the extra-abdominal component of the lipoma and yellow arrows point to the intra-abdominal component of the lipoma Abdomen and pelvis MRI. Sequential sagittal sections in the contrast-enhanced phase: lesion with intra- and extra-abdominal components, hourglass-shaped, narrowing at the passage through the inguinal ring in the abdominal wall. Measures 20 x 11 x 11 cm in the largest diameters (longitudinal x lateral x anteroposterior), at the inguinal ring passage, the "hernia" neck measures 4.1 x 4.4 cm (lateral x longitudinal). Within the lesion, bands of edema are observed, which are enhanced with intravenous gadolinium, while the remainder shows no enhancement. The lesion's volume displaces the midline of the genitalia to the right, compresses and displaces the uterus and bladder to the right inside the abdomen, separates adjacent intestinal loops, compresses and bulges anteriorly the wall of the left hemiabdomen in the hypogastrium, compresses and laterally displaces the left iliac vessels, and has intimate contact with the left iliopsoas muscle.

The case management involved seeking care in the general surgery department, where an MRI of the pelvis and abdomen was recommended. The MRI, with multiplanar and volumetric sequences in T1, T2, and DIFFUSION, with paramagnetic contrast, confirmed the presence of a lipomatous tumor with extraordinary dimensions of 20 x 11 x 11 cm. It was located in the left inguinal canal, extending both intra- and extra-abdominally to the left labia majora (Figure 1).

The surgical approach was planned, initially opting for videolaparoscopic dissection. The introduction of trocars in the left flank (5 mm), supraumbilical (10 mm), and left flank (10 mm) allowed access to the abdominal cavity. During the intervention, a significant intra-abdominal pre-peritoneal bulge was noted, with a loss of anatomical landmarks traditionally used in inguinal hernioplasties. The opening of the parietal peritoneum and delicate dissection of the lipoma from adjacent structures revealed tenuous adhesions (Video 1).

**Video 1:** Laparoscopic visualization and dissection of the lipoma (transabdominal pre-peritoneal technique), reinforcement of the posterior wall with polypropylene mesh after total lipoma resection

**Figure 2:**
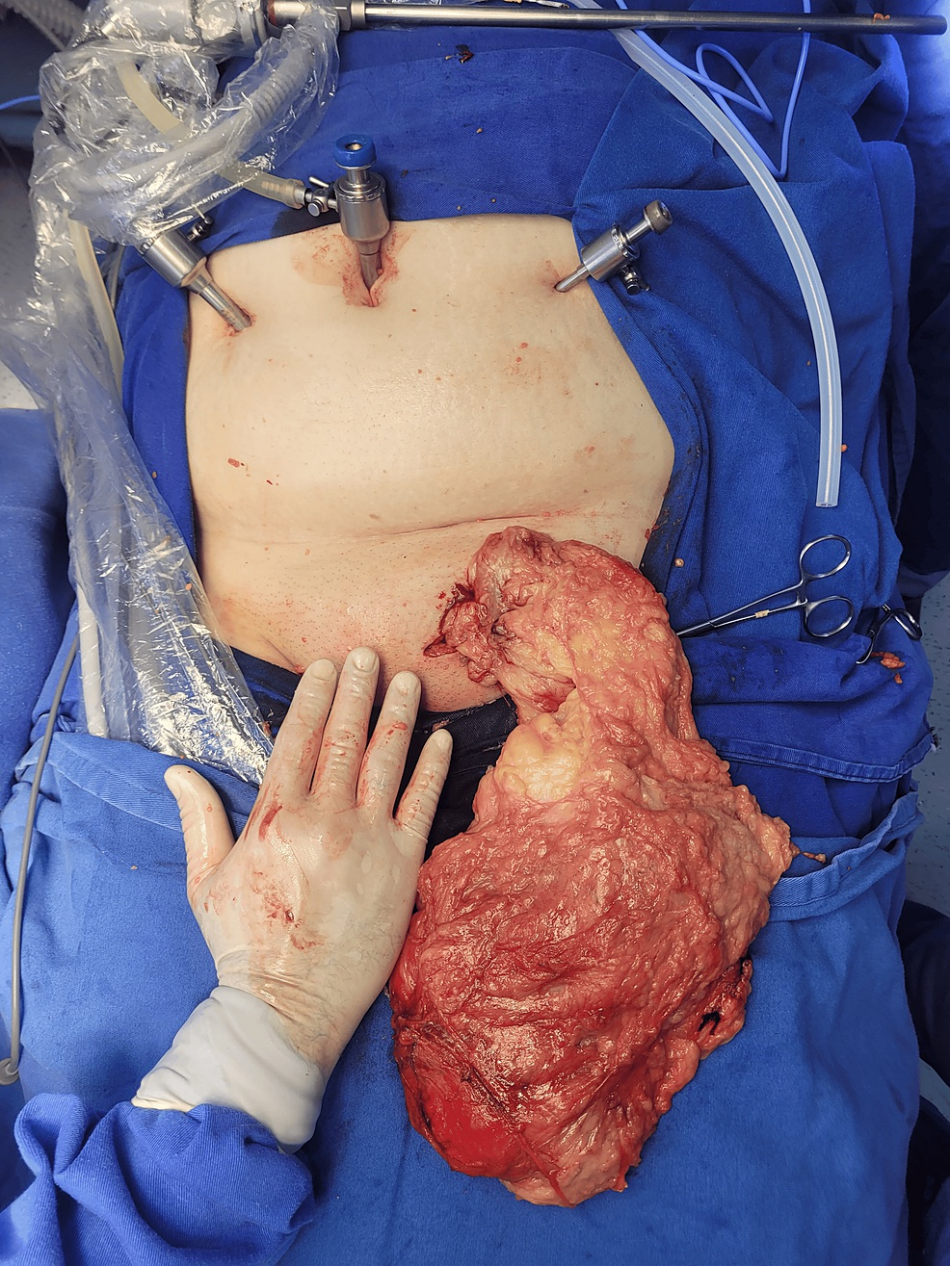
Exposure of the intra- and extra-peritoneal components of the lipoma through left oblique inguinotomy

Subsequently, after detaching the lipoma from intra-abdominal structures, an oblique inguinal incision was made using a conventional technique. This approach allowed the dissection of the distal portion of the lipoma, extending to the left labia majora, culminating in the removal of this lipomatous mass, estimated to weigh approximately 1 kg (Figure 2).

Given the magnitude of the lesion, it was necessary to proceed with the reconstruction of the inguinal anatomy. The abdominal wall was reinforced by applying a polypropylene mesh (15 x 15 cm) posteriorly. Fixation was performed using absorbable endoscopic staples, and the parietal peritoneum was closed with polypropylene suture 3.0. The synthesis of the oblique inguinal incision was done with polyglycolic acid suture 2.0 in the subcutaneous tissue and simple nylon stitches 3.0 on the skin.

**Figure 3:**
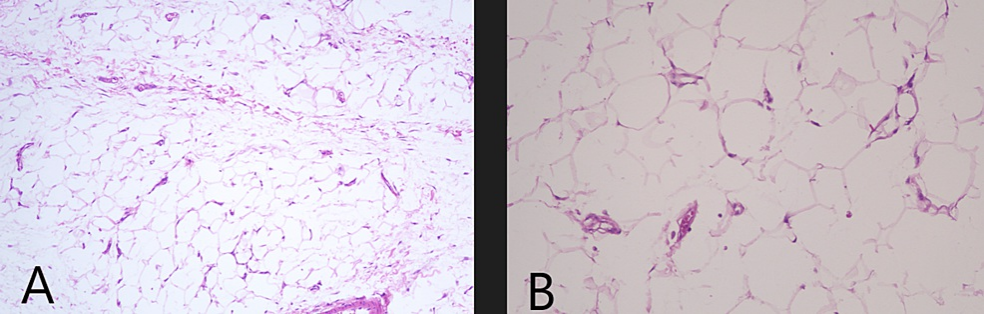
Optical microscopy at 100x (A) and 200x (B) magnification. Hematoxylin and eosin staining. Neoplasm composed of mature adipose cells in a lobular arrangement enveloped by a thin capsule. The cells vary in shape and size, being larger than normal adipocytes. In between, there is a delicate vascular network. Absence of nuclear hyperchromasia

The patient had a favorable postoperative evolution, with discharge on the third day of hospitalization. Subsequent outpatient follow-up over 1 year did not reveal recurrences of the condition. The histopathological examination confirmed the diagnosis of a lipoma without malignant characteristics (Figure 3).

This more detailed description emphasizes the complexity of the case, strategic surgical decisions, and the positive postoperative evolution of the patient.

## Discussion

The high incidence of lipomas in the spermatic cord is a phenomenon widely observed during herniorrhaphy interventions, commonly misclassified as true lipomas in surgical literature [1,2]. However, a more accurate analysis reveals that most of these masses represent extrusions of extraperitoneal adipose tissue in the inguinal canal. A true lipoma in this location is considered a rare occurrence, especially when associated with the possibility of an inguinal hernia, making the distinction between both entities challenging [3,4].

Considering the profile of the patient presented in this report, her uncommon age for the presence of lipomas stands out, as indicated by studies showing an occurrence of only 0.8% in patients under 40 years, being more prevalent in individuals over 60 years [1]. Regarding obesity, although recently disregarded as a risk factor for inguinal hernias [5], it is suggested as a possible predisposition for the development of lipomas in general [6].

Regarding clinical manifestation, the presence of pain and findings consistent with an incarcerated inguinal hernia align with descriptions previously documented in the literature [4,7]. Pain, usually attributed to the compression of inguinal nerves, can manifest chronically, insidiously, or acutely [4,7].

The diagnostic method plays a crucial role, with many lipomas being identified incidentally during herniorrhaphies, regardless of their true nature [1]. The implementation of imaging tests, such as computed tomography and ultrasonography, is essential for an effective diagnosis, providing valuable information in differentiating the lipomatous content associated with inguinal hernias [4,7].

In the context of operative conduct, the literature suggests that only 3% of lipomas with inguinal hernia presentation require the use of combined techniques or conversion from laparoscopic to open surgery. This need usually arises from the inability to reduce the hernia sac or affect the pneumoperitoneum [1]. In cases of diagnostic doubt, a preference for an open technique is supported [4].

Adequate differentiation between true inguinal canal lipomas and non-lipomatous adipose protrusions is crucial to avoiding erroneous treatments and potential complications [4]. It is worth noting the differential diagnosis of liposarcoma [4], requiring a specific surgical approach and neoadjuvant therapy according to established criteria.

This discussion provides a comprehensive analysis of the complexities associated with the diagnosis and management of true inguinal canal lipoma. By integrating current data from the literature, the importance of a multidisciplinary approach and advanced imaging techniques to guide informed therapeutic choices is highlighted. The clinical experience presented in this report adds significant nuances to the understanding of this rare condition, providing valuable insights for everyday clinical practice and guiding future research.

## Conclusions

True inguinal canal lipoma, although rare, demands a careful and precise approach from surgeons. The case presented here, enriched with images and unique clinical details, aims to provide a significant contribution to the understanding and management of this uncommon condition.

The complexity of differentiation between true lipomas and non-lipomatous adipose protrusions underscores the importance of detailed evaluation and accurate diagnosis. The patient's clinical profile, including the less common age range for lipoma development, adds nuances to the understanding of this pathology.

The choice of a combined surgical approach utilizing laparoscopic and conventional techniques demonstrates adaptability and the need for a multifaceted strategy given the substantial dimensions of the lipoma. The reconstruction of the inguinal anatomy and the reinforcement of the abdominal wall highlight a comprehensive approach to ensure positive outcomes and prevent recurrences.

This report not only provides practical insights into the resection of inguinal canal lipomas but also emphasizes the importance of a differentiated consideration of these adipose masses during herniorrhaphy procedures. Emphasizing the correct distinction between true lipomas and similar conditions is crucial to avoid misdiagnoses and complications arising from inappropriate treatment.
